# Exploring the stigma experienced by people affected by Parkinson’s disease: a systematic review

**DOI:** 10.1186/s12889-024-21236-8

**Published:** 2025-01-03

**Authors:** Sophie Crooks, Gary Mitchell, Lisa Wynne, Gillian Carter

**Affiliations:** 1 School of Nursing and Midwifery, Queen’s University Belfast, Belfast, Northern Ireland UK; 2 Parkinson’s Ireland, Dublin, Ireland

**Keywords:** Parkinson’s disease, Stigma, Awareness, Education, Discrimination, Public, Shame, Social stigma, PD

## Abstract

**Background:**

Stigma significantly impacts individuals with Parkinson’s disease (PD) and their caregivers, exacerbating social isolation, psychological distress, and reducing quality of life (QoL). Although considerable research has been conducted on PD’s clinical aspects, the social and emotional challenges, like stigma, remain underexplored. Addressing stigma is crucial for enhancing well-being, fostering inclusivity and improving access to care and support. The review aims to fill this knowledge gap by synthesising existing literature on PD stigma, examining its effects on individuals and families affected, and identifying areas where interventions could reduce stigma’s impact.

**Methods:**

This systematic review was conducted following Joanna Briggs Institute guidance. Studies were identified through searches in six databases, relevant websites, and reference lists. Covidence was used for duplicate removal, screening, and data extraction. Thematic analysis identified key themes from qualitative data, while narrative synthesis integrated findings from qualitative and quantitative studies. The review protocol was registered on PROSPERO (CRD42023399343).

**Results:**

This review included 22 studies published between 2002 and 2024, using both qualitative and quantitative methodologies. Five key themes emerged. The first highlighted stereotypes in PD, such as misconceptions about symptoms, age stereotyping, and supernatural beliefs. The second explored drivers and facilitators of stigma, identifying factors like duration since diagnosis, disease severity, lack of public education, and media representation. The third theme revealed stigma’s impact on mental health and well-being, exacerbating feelings of shame, embarrassment, and social isolation. The fourth, responses and consequences of stigma, detailed strategies employed by individuals with PD to manage stigma, including seeking social support and adopting coping mechanisms. The fifth theme, beyond stigma, explored positive aspects of living with PD, highlighting resilience, positive interactions, and advocacy efforts.

**Conclusion:**

This systematic review underscores the significant impact of stigma on individuals with PD and their caregivers, manifesting as social isolation, diminished QoL, and psychological distress. Key drivers include public misconceptions, cultural biases, and limited awareness and addressing these challenges requires targeted interventions. Recommendations include education to dispel myths, public awareness campaigns and advocacy efforts to reduce stigma, enhance support, and improve QoL.

**Supplementary Information:**

The online version contains supplementary material available at 10.1186/s12889-024-21236-8.

## Background

Parkinson’s Disease (PD) is currently the fastest-growing neurological disorder globally, second only to dementia [1, 2]. Worldwide prevalence has doubled over the past 25 years, now exceeding 8.5 million individuals [3]. It is a progressive neurological disorder due to substantia nigra cell loss, reducing dopamine production [4, 5]. Dopamine deficiency leads to motor symptoms like tremors, bradykinesia, gait issues, and rigidity [6], and non-motor symptoms including speech problems, urinary issues, constipation, sleep disturbances, and neuropsychiatric symptoms [7]. These worsen over time, impacting patients’ quality of life [8]. Symptoms of PD, especially those affecting emotional expression and recognition such as facial masking greatly contributes to stigma [9].

Stigma plays a significant role in the lives of individuals living with Parkinson’s disease as it is a complex social phenomenon encompassing stereotypes, prejudice, discrimination, and exclusion [10]. It differs from shame, which is an emotional response characterised by a deep sense of personal inadequacy or dishonour [11]. While stigma refers to the negative social perception imposed by others due to a particular condition, shame is more personal, stemming from internalised beliefs about one’s own worth or behaviour. Thus, while stigma is shaped by societal attitudes, shame is a self-directed feeling that can compound the challenges faced by individuals with PD [12].

Stigma can manifest in several forms, each affecting individuals in unique ways. Felt stigma refers to the perception that others view a person negatively due to their condition, leading to feelings of anticipated discrimination or exclusion [13]. Enacted stigma, on the other hand, involves actual discriminatory actions or behaviours directed at the individual based on their condition [13]. Affiliated stigma affects caregivers who may experience stigma due to their association with someone living with a stigmatised condition [14]. Lastly, self-stigma occurs when individuals internalise negative beliefs and judgements about themselves, leading to shame, guilt, and a diminished sense of self-worth [15]. These different forms of stigma combine to create complex social and emotional challenges for those affected.

One study [9] exploring stigma in PD reports more than 50% of people with Parkinson’s disease conceal their diagnosis, mask symptoms or avoid appearing in public due to stigma. A major contributing factor to this stigma is the lack of public awareness and understanding of PD and its symptoms, such as tremors, slow movement, and facial masking [16]. These symptoms are often misinterpreted by the public, leading to misconceptions about the capabilities and behaviours of those with PD [16]. Within the context of this review, the public refers to individuals in society who are not directly affected by PD.

As a result of these misunderstandings, individuals with PD and their caregivers frequently face social stigmatisation. This stigmatisation can manifest in various ways, such as being unfairly judged, socially isolated, or excluded from community activities. The impact of stigma on the lives of people with Parkinson’s disease is profound, affecting their quality of life and mental health [17]. Research indicates that stigma in PD can lead to increased levels of depression, anxiety, and social isolation among those living with the disease [18]. Another study [19] highlights the wider health and social impacts of PD stigma, aggravating poverty due to loss of income, increasing severity of disability and sometimes resulting in mortality.

Research on the stigma associated with PD remains limited, despite evidence indicating that stigma can profoundly affect the social and psychological well-being of individuals with Parkinson’s disease. While studies such as Maffoni et al. (2017) [18] and Lubomski et al. (2020) [20] have explored the experience of stigma in PD, their scope and methodologies reveal gaps. Maffoni et al. [18] conducted a review focused solely on qualitative studies, providing insights into patients’ experiences of stigma but limiting broader generalisability and cross-study comparison. Similarly, Lubomski et al. [20] highlighted stigma as a barrier to care, yet primarily within healthcare settings rather than the broader community.

Most existing literature emphasises medical symptoms and treatment options for PD, leaving a lack of comprehensive research into the social and psychological dimensions, including the impact of public and community-based stigma. This review addresses these limitations by employing a mixed-methods approach to synthesise both quantitative and qualitative findings across diverse settings, with a focus on the stigma encountered in everyday social contexts. Filling this gap is essential to developing targeted, evidence-based interventions aimed at reducing stigma and improving the quality of life for individuals with PD and their caregivers.

This systematic review aims to explore PD-related stigma, examining its impact on individuals and their caregivers, and identifying potential interventions to reduce stigma by synthesising existing literature. By understanding the stigma associated with PD, we can determine the extent of the problem and provide recommendations to address it. This will help raise public awareness and promote PD stigma as an important public health issue, ultimately improving the lives of those affected and fostering a more inclusive and supportive society.

This will be achieved through the following objectives; first, to conduct a comprehensive systematic review and synthesise relevant empirical research on PD-related stigma, drawing from multiple databases. Second, to identify recurring themes, patterns, and trends in the literature concerning the experiences of stigma and its impact on individuals affected by PD. Finally, to provide evidence-based recommendations for future research and stigma-reduction strategies, offering targeted interventions to support individuals with PD in addressing and overcoming social stigma. These objectives aim to enhance understanding and inform future efforts to improve the well-being of those impacted by PD.

## Methods

A systematic review was conducted following the Joanna Briggs Institute methodology for Mixed-Methods Systematic Reviews guidance [21]. It is reported in accordance with the Preferred Reporting Items for Systematic Reviews and Meta-Analyses (PRISMA) statement [22] to enhance the quality and transparency of reporting. The review protocol was registered on PROSPERO register of systematic reviews on 13th November 2023 (CRD42023399343) [23]. The PRISMA 2020 Checklist can be seen in Additional File 1.

### Search strategy and selection criteria

Six databases were searched up to August 2024. These were CINAHL, Medline, Embase, PsycINFO, Web of Science and the Cochrane Library. These databases were chosen as their literature focusses on nursing, medicine, psychology, and social sciences. A search of the following websites was also conducted to source relevant grey literature: EThOS, PLOS, NICE and WHO. Reference lists of all included studies were manually searched to identify additional studies not found in the initial search. Internet search engines Google (https://www.google.co.uk/) and Google Scholar (https://scholar.google.com/) were extensively searched for peer-reviewed publications. The search was supplemented by journal alerts from Parkinson’s specific journals such as Journal of Parkinson’s Disease and Parkinsonism and Related Disorders.

Keywords used to conduct the search were based on three main dimensions: Parkinson’s disease, stigma (and related terms) and public perception. These keywords were carefully selected to capture a broad range of articles related to Parkinson’s disease and the associated stigma. Using variations of “Parkinson’s disease” ensured that different terminologies that may be used in academic literature were covered, while the terms related to stigma and discrimination were chosen to encompass the different dimensions of stigma that individuals with PD may experience. Terms related to public perception helped to identify articles that examined societal views and misconceptions, and discussed the social dimensions of living with PD. The truncation wildcard (*) was used on key words, allowing alternate forms of the word to be searched [24]. All search terms and Boolean logic can be seen in Additional File 2.

### Identification of studies

In this review, research exploring the stigma experienced by people affected by PD was of interest. Research incorporating people with Parkinson’s disease and their carers were included. Carers often share in the challenges of PD, therefore they were included to provide a comprehensive view of stigma’s impact on both individuals with PD and those who support them. In studies where the experiences of people with Parkinson’s disease was examined alongside other conditions, data were extracted only about PD without limitations. No data limitations were put in place during database searches. A summary of the inclusion/exclusion criteria used is provided in Table 1.

**Table 1:** Inclusion/exclusion criteria

Inclusion	Exclusion
• Empirical research on the experiences of people living with Parkinson’s disease or their carers • Grey literature including thesis, dissertation, or full text of conference abstracts • English articles	• People who do not have PD or people who are not carers for Parkinson’s disease • Non-empirical research • Commentary papers • Literature reviews/systematic reviews (excluded but reference lists searched for potential papers) • Studies about other neurological conditions not including PD

### Study selection

The systematic review software Covidence was used to assist in the removal of duplicates, screening, and data extraction [25]. Studies were imported and duplicates were removed. The inclusion and exclusion criteria were used to screen papers within Covidence and determine relevance to this review. Stage 1 of screening involved examining studies by title and abstract, with the primary screening conducted by SC. To enhance reliability, a sample of 25% of studies was also checked by GC, serving as a secondary reviewer to reduce potential bias and improve consistency in selection. The 25% sample size was chosen as a manageable proportion for cross-checking while providing a robust reliability measure across the dataset. Stage 2 involved screening of full text studies by SC and GC independently. At this stage, both researchers (SC & GC) screened 100% of full text studies. Conflicts throughout the screening process were resolved following discussion with GM.

### Quality appraisal

The quality of papers included in the review was appraised using the Joanna Briggs Institute (JBI) Critical Appraisal Tool. Qualitative studies were evaluated with the JBI checklist for qualitative research [26], while quantitative studies were assessed using the JBI checklist for analytical cross-sectional studies. Covidence software was used to manage and streamline the appraisal process, allowing reviewers to independently assess each study. In cases where there were disagreements between reviewers, a third reviewer was consulted to resolve discrepancies, ensuring an unbiased evaluation. To further enhance reliability, inter-rater agreement was calculated using Cohen’s kappa [27], which provided a quantitative measure of consistency between reviewers. Twelve studies were deemed to be of high quality [28–39] and the remaining ten were medium quality [19, 40–48]. No studies were deemed as low quality, therefore were not excluded.

### Data synthesis and analysis method

This review adopts a mixed methods approach, encompassing both qualitative and quantitative studies. Narrative synthesis, as defined by Popay et al. (2006) [49], involves using language and text to summarise and present findings across multiple studies, adopting a textual or ‘storytelling’ approach. It is a robust scientific approach that facilitates the integration of both quantitative and qualitative data related to a specific phenomenon [50]. Narrative synthesis was used for this systematic review and was conducted by the researcher (SC).

In this systematic review, both qualitative and quantitative methods of analysis were employed to provide a comprehensive synthesis of the included studies. For the qualitative component Braun and Clarke’s (2022) thematic analysis [51] was used to systematically identify and categorise recurring patterns and themes within the findings. This involved coding the qualitative data from each study, organising these codes into broader themes, and refining these themes using NVivo 11.

For the quantitative studies, the findings were converted to comprise textual descriptions or narrative views of quantitative results, allowing for a more holistic understanding of the data. This conversion process enabled the integration of quantitative findings into the broader narrative framework of the review. The integration of qualitative and quantitative data was achieved through a mixed methods synthesis, following the JBI methodology for Mixed-Methods Systematic Reviews [52]. This approach allowed the triangulation of the data, enhancing the robustness and validity of our conclusions.

## Results

### Study characteristics

The review PRISMA (2020) [22] flowchart is presented in Additional File 3. A total of 5441 studies were imported into Covidence and 1190 duplicates were removed. The inclusion and exclusion criteria were used to screen papers within Covidence and determine relevance to this review. Firstly, 4252 studies were screened by title and abstract (SC) and a sample checked (GC). In total, 4206 studies were determined irrelevant for this review, leaving a remaining 46 studies for full text screening. In total, 24 studies were excluded due to providing outcomes not relevant to the review (*n* = 15), interventions based on disease management (*n* = 2), the population did not focus on PD (*n* = 2), and not empirical research (*n* = 2). Literature reviews (*n* = 3) were also excluded however, reference lists were screened for papers that may not have shown during the database search that may be suitable for the review. During the reference list search, one study was determined suitable for the review after title, abstract and full text screening. A final total of 22 papers were deemed suitable for this review. A full PRISMA flow chart can be seen in Additional File 3.

This review included 22 research studies published between 2002 and 2024. Of these, 11 used a qualitative design, and the remaining 11 employed a quantitative approach. Eleven studies in the review had a cross-sectional design using questionnaires, one using a cohort design via a 3-year prospective study [37] and 11 used a qualitative approach using interviews and focus groups. A table of study characteristics can be seen in Additional File 4.

The included studies took place in various geographical locations: seven studies in the USA [34, 36–38, 41, 42, 47, 48], four in the UK [33, 39, 44, 46], two in China [35], two in Brazil [32, 45], and one each in Mexico [34], Israel [28], Sweden [40], Kenya [19], Tanzania [29], Jordan [30], Taiwan [31] and France [43]. The studies were conducted in diverse settings, including participant homes, clinics, cafes, PD support groups, hospitals, academic medical centres, and online platforms. The geographic diversity of these studies suggests that the results may be shaped by differences in healthcare systems, cultural beliefs, and participant characteristics, all of which could affect the outcomes and limit the generalisability of the findings.

Sample sizes ranged from six [46] to 362 participants [48] with a combined total of 2,502 participants. Studies included samples of people with Parkinson’s disease [19, 29–33, 35–41, 43–48], spouses/carers of people with Parkinson’s disease [19, 28, 29, 31, 34, 41], healthcare workers and traditional healers [29]. All studies included both female and male participants, apart from one study [40] that only included female participants. The mean age of participants in the included studies was 65.1 years old, with the range being 30–94 years old. Mean disease duration of participants was 8 years. The variability in sample sizes across studies may introduce bias, as smaller samples could result in less reliable or less generalisable findings. Additionally, studies with larger samples may overrepresent certain characteristics, skewing the results. This variation was considered when interpreting the overall findings as it could influence the accuracy and applicability of the conclusions drawn from these studies.

All 22 studies reported obtaining ethical approval from appropriate review bodies. In total, 21 studies included information about informed consent processes. One study by Hermanns (2013) [42] did not include information about obtaining informed consent from participants for the study. This omission of informed consent can raise ethical concerns and lead to potential bias as participants may not fully understand their involvement in the study. Six studies reported conducting the studies in accordance with the Declaration of Helsinki (1964) [19, 31, 32, 34, 38, 48, 53], and six maintained confidentiality and anonymity throughout the study [28, 29, 31, 40, 41, 46]. Three studies reported voluntary participation [28, 30, 40]. A thorough review of included studies in this review revealed no significant ethical concerns.

### Data collection

#### Qualitative studies

Ten papers adopting a qualitative approach used individual interviews to collect data [19, 29, 30, 40–46]. Of these, some combined interviews with other methods such as focus groups [29, 41], observations [42, 43], and questionnaires [31]. One study by AboJabel et al. (2021) [28] used only focus groups for the data collection method.

The qualitative data collection took place in a variety of settings including participant homes [19, 40, 43], clinics and cafés [19] and PD support groups [42]. Seven studies did not mention where interviews took place [28–30, 41, 44–46]. Use of an interview guide was discussed in six studies [28–30, 41, 43, 46], four of which included the interview guide within supplementary material [28, 30, 41, 43]. Researchers conducted the interviews in seven studies [19, 28–30, 41, 43, 44]. The remaining qualitative studies did not mention who conducted the interviews within the studies.

#### Quantitative studies

All eleven quantitative studies included in the review used questionnaires to collect data. Three collected data via an online questionnaire [33, 36, 39], two studies collected questionnaire data in hospitals [32, 35], one combined both [31], and one in a public academic medical centre [34]. These studies used self-completion questionnaires for data collection. The remaining four studies did not mention by what means the questionnaire data were collected [37, 38, 47, 48].

Across the studies, 24 different outcome measurements were used. The Parkinson’s Disease Questionnaire (PDQ-39) [54] was the most common instrument, being used by seven studies [32, 36–39, 47, 48]. This questionnaire includes 39 items assessing how often people with Parkinson’s disease experience difficulties across eight dimensions of daily living including relationships, social situations and communication. It also assesses the impact of Parkinson’s on specific dimensions of functioning and wellbeing. Of these studies, six used PDQ-39 and one [39] used PDQ-8 which is a shorter version with eight items. Both versions of the PDQ are validated instruments that have been widely used for assessing the quality of life for people with Parkinson’s disease.

Several other validated scales were used in the studies reviewed to assess various aspects of PD. The most common scales are shown in Additional File 5.

### Study results

Through the process of data analysis and synthesis, five distinct themes emerged. The first theme, *stereotypes in Parkinson’s disease*, examines the common misconceptions, preconceived notions, and prevailing stereotypes surrounding individuals with PD within the broader societal context. The second theme, *drivers and facilitators of stigma*, explores the underlying societal, cultural, and psychological mechanisms that foster stigmatising attitudes and behaviours towards individuals living with PD. The third theme addresses *the impact of stigma on mental health and well-being*, including feelings of embarrassment and shame associated with the stigma of PD. The fourth theme, *response and consequences of stigma*, considers the strategies adopted by those with PD to cope with public stigma. Finally, the fifth theme, *beyond stigma*, sheds a more positive light on the impact of living with PD, offering a comprehensive understanding of the condition.

## Theme one: stereotypes in Parkinson’s disease

Across all studies, participants highlighted the prevalence of stereotypes and misconceptions about PD in the public domain [19, 28–48]. These misconceptions often led to stigmatising behaviours, such as avoidance and discrimination, directed toward individuals with PD and their caregivers.

### Visible symptoms as the defining characteristic

One prevalent stereotype is the misconception that PD is defined solely by visible motor symptoms, particularly tremor [28]. Participants expressed frustration over this oversimplification, emphasising the lack of awareness regarding other symptoms of PD, particularly non-motor symptoms. For instance, one study [28] reported that participants noted the public’s limited understanding of non-motor symptoms such as speech disorders and muscle stiffness, which undermines the complexity of PD.

*“I think it (the perception of the illness) is overly related to the tremors. Nobody understands the suffering caused by muscle stiffness” (* [28] *p.3)*

The literature also indicated that difficulties with activities of daily living (ADLs) and motor symptoms were associated with higher levels of stigma in individuals with PD. One study [32] found that ADL difficulties contributed to greater stigma, and another [33] linked independence in ADLs to lower levels of stigma. Lin et al. (2022) [37] demonstrated that impairment in ADLs, motor examination scores, and non-motor symptoms correlated with higher levels of self-stigma. Furthermore, Ma et al. (2019) [47] noted that difficulties with facial expression, a common PD symptom, tended to increase feelings of stigma and reduce quality of life. Another study [35] found significant associations between motor symptoms and both self-stigma and enacted stigma.

### Age stereotyping and misconceptions

Another common stereotype was the belief that PD exclusively affected older individuals [19, 28, 29, 43, 44]. This ageist stereotype was perceived to lead to delayed diagnoses and misinterpretation of symptoms, particularly in younger individuals. Participants reported encountering disbelief or scepticism from others upon disclosing their diagnosis, often leading to concealment of their condition to avoid judgement [28, 43]. Healthcare professionals’ biases further contributed to perceived ageist stereotypes and delayed treatment [46]. Media portrayals emphasising advanced-stage symptoms also were noted to skew public perceptions of PD.

*“Everyone stands there not understanding how such a thing can occur (the disease befalling a young person).” (* [28]; *p. 3).*

Quantitative research supported these findings, with two studies [32, 35] reporting associations between age and stigma, particularly among females. One study [33] found that older individuals reported lower levels of perceived and experienced stigma than younger individuals, while another study [48] identified younger age as a predictor of self-perceived stigma, especially among men.

### Supernatural beliefs and alternative explanations

Supernatural beliefs, including witchcraft or curses, were also identified as alternative explanations for PD symptoms [19, 29]. Participants in these studies reported that such beliefs contributed to the stigmatisation of individuals with PD, portraying them as afflicted by malevolent forces or feigning symptoms, leading to social isolation and discrimination. These misconceptions were felt to not only undermine the understanding of PD but also exacerbate the stigma and challenges faced by those living with the condition.

*“people think [the person with Parkinson’s] is bewitched or they think she is pretending or she’s drunk” and “there’s no way you’re going to convince such people that this person is just sick. The most meaningful [explanation] is witchcraft” (* [19]; *p. 5)*

## Theme two: drivers and facilitators of stigma

Stigma surrounding PD was shaped by various drivers and facilitators, contributing to negative public perceptions and attitudes toward individuals affected by the condition.

### Time since diagnosis and disease severity

Stigma experiences in individuals with PD were noted to be influenced by time since diagnosis and disease severity. One study [33] found that as the number of years since diagnosis increased, individuals reported higher levels of felt, enacted, and total stigma, which suggested more pronounced stigma in later stages. Hou et al. (2021) [35] also found disease duration to be significantly associated with stigma in PD with Stigma Scale for Chronic Illness (SSCI) score (*p* = 0.003) and felt stigma (= 0.004), particularly in males (p = < 0.001). Additionally, Ma et al. (2016) [38] found that all stigma measures correlated with disease severity, with more severe PD being linked to increased stigma and poorer quality of life (p = < 0.05). Conversely, Salazar et al. (2019) [48] found no significant contribution of disease characteristics to stigma perception, indicating a complex and multifaceted experience of stigma in PD.

### Presence of additional health conditions

Comorbidities, especially mental health issues, exacerbated stigma in PD patients. One study [36] reported that 79% of PD patients had additional health conditions, such as thyroid disease, depression, and anxiety, which correlated with higher stigma levels and lower quality of life. They demonstrated that depression and anxiety showed strong positive relationships with stigma (*p* < 0.001). Another study [35] also found significant associations between anxiety, depression, and stigma with higher SSCI scores (p = < 0.001) and higher felt stigma (*p* = 0.001). These results showed these emotional challenges are closely linked to how much stigma people feel. This study also measured affiliate stigma, which refers to the stigma experienced by individuals who are closely associated with someone who has a stigmatised condition, such as a caregiver [55]. Mental health issues were significant predictors of affiliate stigma (*p* = 0.187) [34], highlighting their impact on stigma in PD.

### Lack of public awareness and education

A prominent driver of stigma identified in the review was the lack of awareness and education about PD within communities [19, 29, 42–44]. Participants noted that misconceptions and misinformation about PD led to stigmatisation. One study [43] emphasised the absence of public information about PD, resulting in stereotypes and stigma. Another [45] reported that many people were unaware of what PD entailed, perpetuating misconceptions and hindering early diagnosis and intervention. Similarly, one participant from Valcarenghi et al. (2017 p. 275) [45] remarked:

*“One thing I’ve learned is that many people do not know what Parkinson’s disease is.“(* [45]; *p.275).*

### Misattributions and cultural beliefs

Misattributions of PD symptoms to aging, clumsiness, or cold weather contribute to stigmatisation [19, 29, 44, 46]. Cultural beliefs, such as witchcraft or curses, further exacerbated stigma.

*“There’s that whole thing of whatever you don’t understand is witchcraft.Some spirits are behind this whole thing” (* [19]; *p.5)*

Participants in Fothergill-Misbah’s (2023) [19] study reported cultural beliefs as drivers of stigma, with some attributing PD to supernatural causes. These beliefs, often linked to blame and punishment, notably impacted individuals with PD, leading to social isolation and discrimination. Shah et al. (2022) [44] highlighted the role of cultural ideologies in stigmatising PD, with participants reluctant to disclose their condition due to associated shame or taboo.

*“And there is a stigma attached to the condition back home*,* where I come from. Wherein*,* no one tends to divulge–well actually it’s a private information*,* like my condition. So*,* nobody would like*,* even my family wouldn’t like to tell anybody else that I’m having this condition. So*,* it’s better off taking me as a drunk*,* rather than a PD patient.” (* [44]; *p. 11).*

### Public perception and media representation

Media representation was noted to influence public perception of PD, often focusing on advanced-stage symptoms and celebrity narratives [43]. This skewed portrayal led to misconceptions and further stigmatised individuals with PD. Two studies [19, 43] noted the absence of media representation and public awareness about PD, highlighting the need for better public education to reduce stigma.

*“I’ve never heard anybody*,* [Parkinson’s has] never been featured in the newspapers*,* no doctors talk about it the way they talk about anything else. There is no awareness about it at all”. (* [19]; *p. 5).*

In summary, stigma surrounding PD is driven by factors such as time since diagnosis, disease severity, comorbidities, lack of public awareness, cultural beliefs, healthcare system limitations, and media representation. Addressing these issues through public education, improved healthcare training, and accurate media portrayal can help mitigate stigma and improve the quality of life for individuals with PD.

## Theme three: impact of stigma on mental health and well-being

Stigma surrounding PD was seen to considerably impact the mental health and well-being of affected individuals, exacerbating feelings of shame, embarrassment, and social isolation.

### Feelings of shame and embarrassment

Stigma induced profound feelings of shame and embarrassment among individuals with PD, as highlighted in several studies [40, 41, 45]. Participants expressed concerns about being perceived as incompetent, leading to self-stigmatisation and a desire to conceal their condition. For example, one participant in Caap-Ahlgreen et al. (2002) [40] stated:

“*People around me think I am incompetent; think I am an idiot” (* [40]; *p. 90).*

Another participant in one study [41] expressed embarrassment about her symptoms:

“*One person did see me shake and go*,* ‘Oh my god*,* I hope it’s not Parkinson’s’” (* [41]; *p. 64).*

This sense of shame often resulted in social withdrawal, avoidance of public spaces, and diminished self-esteem, worsening mental health.

Stigma also contributed to the loss of independence for individuals with PD, further exacerbating feelings of shame. Participants in one study [46] discussed struggles with maintaining autonomy, with one stating:

“*The loss of my independence was*,* very very hard to bear” (* [46]; *p. 293).*

This loss of independence was noted to intensify the emotional burden and contributed to a diminished sense of identity and self-worth [46].

### Impact on social and work life

Stigma associated with PD extended to social networks and the workplace, worsening feelings of self-stigma and isolation. Participants reported discomfort among friends, family, and colleagues due to PD’s physical symptoms, leading to self-consciousness and avoidance of social interactions. One study [41] noted unease among friends and family members, contributing to participants’ social withdrawal. In another study [44], participants avoided public spaces due to embarrassment, with one stating:

“I tend to stick to my room, rather than go anywhere. I feel a little bit embarrassed as well to go out because I feel low” ([44]; p. 10).

In the workplace, individuals with PD faced stigma from co-workers, leading to self-stigmatisation and reluctance to disclose their condition. One participant [41] expressed frustration about explaining the disease at work, wishing for a badge that says, “don’t ask, I have Parkinson’s disease” (p. 64). This fear of judgment and discrimination was seen to diminish self-confidence and to contribute to frustration and isolation.

Stigma also leads to social withdrawal and isolation, contributing to feelings of loneliness and depression among individuals with PD [19, 30]. Participants described withdrawing from social activities due to fear of judgment, which then perpetuated feelings of depression and anxiety.

## Theme four: response and consequences of stigma

Despite the challenges posed by stigma, individuals with PD employed various strategies to manage its impact on their lives, including seeking social support, engaging in advocacy efforts, and adopting adaptive coping mechanisms to navigate social interactions and mitigate stigma [44, 46].

### Response strategies to stigma

Individuals with PD used diverse strategies to cope with public stigma, from concealing symptoms to avoiding social situations. Participants described efforts to hide their symptoms in public, such as holding onto purse straps or keeping hands in pockets to conceal tremors [42]. Some individuals even relocated to escape scrutiny, with one participant noting:

*“Sold the house. Would be good to leave this area” (* [40]; *p. 93).*

Avoidance strategies, like restricting social activities to essential events only, were also common, especially among women. One participant explained:

*“The disease has affected my social life hugely as I get embarrassed because of the symptoms. I do not leave home unless it’s very necessary” (* [30]; *p. 76).*

### Consequences of stigma on social interactions

Stigma deeply impacted social interactions, contributing to social isolation and withdrawal from community engagement [19]. Participants reported feeling watched and judged, leading to heightened self-consciousness and discomfort in public spaces [30]. Workplace stigma further exacerbated social isolation, with individuals reluctant to disclose their condition due to fear of negative reactions from colleagues [19]. This avoidance of public spaces and social interactions resulted in a diminished quality of life. One participant reported:

*“Over the last year*,* I’ve become more withdrawn. I don’t want people to see me. I’m afraid to see how people look at me” (* [43]; *p. 16).*

### Psychological consequences of stigma

The psychological impact of stigma on individuals with PD was profound, leading to feelings of shame, embarrassment, and low self-esteem [30, 40]. Participants described internalised stigma from societal beliefs about their capabilities, leading to feelings of inadequacy and worthlessness [19]. Depression, anxiety, and loneliness were common, exacerbated by public stigma [30]. Stigma-induced psychological distress hindered individuals’ ability to seek social support and engage in meaningful activities, perpetuating a cycle of social isolation and diminished well-being.

Self-compassion plays a crucial role in mitigating the negative impact of stigma on mental health. One study [33] found that higher levels of self-compassion were associated with lower levels of depression, anxiety, and stress, both independently and through a significant indirect effect on felt stigma (p = < 0.001). However, self-compassion did not moderate the relationship between enacted stigma and mental health outcomes (p = < 0.00). Emotional well-being was significantly associated with stigma (*p* = 0.12) (da Silva et al., 2020), and mental health symptoms contributed to experiences of stigma (*p* = 0.187) [34]. Individuals with PD who experience stigma tended to have more severe depression and poorer quality of life (p = < 0.05) [38]. Similarly, one study [31] also found higher stigma severity to be an associated factor of depression in both people with Parkinson’s disease (*p* = 0.001) and their caregivers (*p* = 0.006). Verity et al. (2020) [39] highlighted the significant impact of stigma on health-related quality of life and depression (*p* = 0.01), underscoring the complex interplay between stigma, self-compassion, mental health, and quality of life in individuals with PD.

## Theme five: beyond stigma

While the stigma surrounding PD is undeniable, it is essential to recognise that not all attitudes towards PD are negative. In this fifth and final theme, the nuanced aspects of the PD experience beyond stigma are explored, highlighting positive interactions, resilience, and advocacy efforts among individuals living with the condition.

### Positive interactions and support

Despite the pervasive stigma surrounding PD, individuals often encountered positive interactions and support from their social networks and communities. Chiong-Rivero (2011) [41] highlighted instances where individuals received assistance and empathy from strangers in public settings, challenging preconceived notions of stigma. For example, one participant described how bystanders on a bus rushed to help after she tripped, illustrating compassion and support from unexpected sources [41]. Similarly, Fothergill-Misbah [19] noted that despite the challenges of stigma, individuals with PD often found solace and support in peer-led support groups. These groups were felt to serve as safe spaces where the individual could share their experiences without fear of judgment, which fostered a sense of community and understanding for them.

### Resilience and advocacy efforts

Stigma was also reported to catalyse resilience and advocacy efforts among individuals with PD. Despite facing discrimination and misunderstanding, some participants in Fothergill-Misbah [19] expressed a desire to educate their communities and raise awareness about PD. Additionally, Hermanns [42] highlighted the capacity of individuals to accept themselves for who they were, embracing their identity as people living with PD. This self-acceptance represented a powerful form of resilience in the face of societal stigma and discrimination.

## Discussion

Research addressing the stigma faced by individuals with PD in various social contexts and cultural settings is relatively recent. Of the 22 studies included in the review, 17 were conducted in the last decade, suggesting an increasing focus on this issue. The findings reveal significant public stigma towards individuals with PD across various global contexts, including Tanzania, Jordan, the United States of America (USA), and the United Kingdom (UK). This widespread stigma demonstrates its universal nature, transcending cultural and socioeconomic boundaries. Understanding this global perspective is crucial for developing comprehensive strategies to address the pervasive impact of stigma. This has been demonstrated in one study [56] who found raising awareness in various global contexts can combat stigma associated with COVID-19.

One key aspect of the review highlighted by the inclusion of various countries is the diverse cultural beliefs and alternative explanations for PD which majorly contributes to stigma. Multiple studies [19, 29, 44, 46] highlighted that PD is often misattributed to aging, clumsiness, or supernatural causes. These misconceptions lead to shame, embarrassment, and reluctance to disclose the diagnosis, further exacerbating stigma. This includes the belief that causes such as nature spirits, poverty, personal hygiene, and violation of social taboos. Such beliefs, common in indigenous communities, may influence health-seeking decisions where individuals may prioritise traditional healing practices, which reflect deeply valued cultural perspectives on health, before considering medical care [19, 29, 57]. This has been seen in other diseases such as cancer, where seeing healers may potentially affect timing of diagnosis and treatment in indigenous communities [58].

Additionally, these cultural beliefs and fears greatly influence public perception and treatment of individuals with PD. This is similar to other diseases like dementia, where in some cultures witchcraft, bewitchment and evil spirit possession are seen as causes of the disease and can lead to stigma [59].

A significant manifestation of PD stigma is the stereotype that it exclusively affects older individuals. This belief can lead to younger individuals feeling profound shame about their diagnosis, causing them to hide their condition and avoid seeking necessary support and resources [28]. The age-related stereotype also affects healthcare professionals, who may attribute PD symptoms to aging rather than recognising them as disease manifestations. Such assumptions by healthcare providers perpetuate stigma and contribute to the mismanagement of the disease, negatively impacting patients’ health outcomes and quality of life. This attribution of disease to older age is also common in Multiple Sclerosis (MS), as one study found younger individuals living with MS encountered more stigma [60] which indicates that disease stigma affecting younger individuals is prevalent across various conditions.

A primary physical manifestation of PD stigma is the public’s tendency to focus on visible symptoms, such as tremors, while neglecting non-visible aspects like cognitive impairment and mood disorders [28]. This narrow perception reduces the disease to its apparent features, leading to inadequate support and heightened emotional burdens for individuals with PD and their caregivers. Motor symptoms and difficulties with activities of daily living (ADLs) contribute significantly to public stigma, particularly for those with noticeable impairments like lack of facial expression [47]. This stigma can exacerbate motor symptoms through increased psychological stress and social isolation, further burdening caregivers [38]. Similarly, people living with MS frequently experience significant stigma due to the visible symptoms of the disease, as evidenced by two studies, highlighting the impact on their quality of life [61, 62].

Stigma also profoundly impacts the mental health of individuals with PD, causing feelings of shame, embarrassment, social withdrawal, and worsened mental health [19, 40]. This is similar across multiple conditions including dementia [62], cancer [63] and MS [64], causing low self-esteem, isolation and poor mental health in these conditions. This can have detrimental impacts on the well-being of individuals and their caregivers, who face significant emotional and psychological stress due to the demanding nature of living with disease and caregiving in a stigmatised context [65].

Stigma in the workplace was a common theme in this review, leading to a reluctance in disclosing the condition, resulting in diminished self-confidence, loneliness, and depression [19, 41]. This can severely impact careers and finances, creating additional stress for caregivers [66]. This is also prevalent in other diseases like MS, where one study found 79.2% of people with MS have experienced workplace stigma [67]. This resulted in people feeling greater stress, low self-esteem and lower QoL.

The review has shown how stigma increases with the duration and severity of PD. Eccles et al. [33] noted higher levels of stigma among individuals with longer disease durations. Additionally, Ma et al. [38] found that as PD progresses, so does the stigma, particularly due to visible symptoms such as tremors, balance difficulties, and wheelchair use. These symptoms lead the public to focus on motor impairments, neglecting broader impacts like cognitive impairment and mood disorders. This is similar in other illnesses like cancer, where time since diagnosis and disease severity have shown to have a positive effect on stigma experienced by the public [68]. This narrow perception is problematic as it fosters a one-dimensional view of PD, marginalising those affected.

A significant factor in PD stigma is the lack of public education and awareness. Multiple studies [19, 29, 34, 42–44] emphasised that poor understanding of PD reinforces stereotypes, hinders early diagnosis and treatment, and leads to social withdrawal and mental health issues. A well-informed public can better understand the full spectrum of PD, including both its visible and non-visible symptoms. By dispelling myths and correcting misconceptions, education can reduce the stigma associated with PD [69]. This has been demonstrated in a study by Jagota et al. (2020) [69], where a public social media educational campaign showing videos of people with Parkinson’s disease had a positive impact in creating awareness about PD.

Poor media representation also impacts PD stigma. One study [43] noted that media coverage often focuses on advanced-stage symptoms, creating a skewed and frightening image of PD. This reinforces stereotypes and stigma, as seen when a BBC drama inaccurately described PD as a ‘terminal condition,’ leading to complaints from viewers [70].

Furthermore, the lack of comprehensive media representation means that the diverse experiences of those with PD are not adequately portrayed. The media’s depiction of PD is often limited to high-profile cases like Michael J. Fox, who serves as a spokesperson for the disease. While his advocacy is vital, it also highlights the scarcity of broader representation, which is necessary for a nuanced public understanding of PD.

This inaccurate media representation creates a distorted and negative public perception [46]. This skewed portrayal reinforces stereotypes, heightening public fear and misunderstanding about PD. As a result, individuals with PD may find it challenging to integrate into society and receive empathy and support.

This negative image of disease portrayed by the media has also been seen in dementia. One study [71] discussed common frames of the disease used by the media, including being ‘old’, being a burden of care, and the ‘living dead’ frame, where common descriptions include ‘death before death’. This intense media scaremongering approach can lead to social stigmatisation and create a social distance between those with dementia and those without.

Comprehensive awareness campaigns, such as those used within the aforementioned study [69], using various media platforms can disseminate accurate information about PD. Additionally, integrating PD education into school curricula and workplace training programs can normalise discussions about the disease and encourage inclusivity. These programmes can equip young people and professionals with the knowledge to support peers and colleagues with PD and have proven to be successful in previous research where education in schools has positively changed students’ knowledge about and attitudes towards dementia [72–74]. Utilising the influence of public figures such as Michael J Fox and ensuring accurate media portrayals can also help shift perceptions, presenting balanced views of PD across various stages and backgrounds. By addressing these factors, it is possible to reduce stigma and improve the overall well-being of those living with PD.

These targeted interventions mentioned could help mitigate cultural beliefs surrounding PD. For instance, awareness campaigns aimed at disseminating accurate information are essential, particularly in indigenous communities where individuals may avoid medical care and turn to traditional healers. By providing reliable information, these campaigns can challenge misconceptions, such as the belief that PD is caused by witchcraft, thereby reducing cultural stigma and encouraging earlier medical intervention.

## Strengths and limitations

A key strength of this systematic review is that, to our knowledge, it is the first to comprehensively explore the stigma experienced by individuals affected by Parkinson’s disease. The review used an extensive search strategy, covering a wide array of databases, including Google Scholar and reference lists, combined with broad search terms and well-defined inclusion and exclusion criteria. This approach maximised the capture of relevant studies to address the research question. However, a notable limitation is the restriction to English-language publications, which may have excluded valuable insights on stigma from non-English-speaking regions, potentially limiting the understanding of stigma experiences across different cultural contexts.

## Implications

This review highlights implications for policymakers, clinicians, and researchers. Policymakers should prioritise public awareness campaigns to correct misconceptions about PD and reduce cultural and age-related stigma. Clinicians should adopt a holistic, culturally sensitive approach that addresses both the physical and psychological effects of stigma in PD, offering support for mental well-being alongside medical treatment. Future research should focus on how cultural beliefs shape stigma, as well as on the resilience and advocacy demonstrated by those living with PD, to inform more empowering and inclusive healthcare strategies.

## Conclusion

This systematic review examined the multifaceted nature and far-reaching impacts of stigma surrounding PD. The review synthesised findings from diverse studies, and revealed that stigma manifests through negative stereotypes, misconceptions, social exclusion, and discrimination, affecting both individuals with PD and their caregivers. The underlying drivers include lack of awareness, cultural beliefs, and societal norms, which perpetuate misconceptions and hinder access to essential resources and support. The consequences are profound, leading to social isolation, reduced quality of life, and psychological distress, as well as challenges in employment, financial stability, and healthcare access. However, the review also highlighted resilience, advocacy, and positive interactions, underscoring the agency and strength of individuals with PD. These insights highlight the need for targeted education, awareness campaigns, and advocacy initiatives. Future research should focus on longitudinal studies assessing the effectiveness of stigma-reduction interventions and explore how cultural and societal factors influence stigma. Such studies could fill gaps in the current literature and provide evidence-based strategies to improve the well-being of individuals with PD and their caregivers.

## Electronic supplementary material

Below is the link to the electronic supplementary material.

Supplementary Material 1

Supplementary Material 2

Supplementary Material 3

Supplementary Material 4

Supplementary Material 5

## Data Availability

All data generated or analysed during this study are included in this published article and its supplementary information files.

## Abbreviations

PD: Parkinson’s Disease
QoL: Quality of Life
PI: Parkinson’s Ireland
JBI: Joanna Briggs Institute
