# Supplementary material for: Exploring the stigma experienced by people affected by Parkinson’s disease: a systematic review
Source: BMC Public Health. 2025 Jan 3;25:25. doi: 10.1186/s12889-024-21236-8 (PMC11697948; doi:10.1186/s12889-024-21236-8)
Supplement: Supplementary file 2 — Supplementary Material 2 [file 12889_2024_21236_MOESM2_ESM.docx]

| **Parkinson’s Disease** | **Stigma** | **Public Perception** |
| --- | --- | --- |
| - Parkinson’s disease - Parkinson disease - Parkinson* - PD | - Stigma* - Stigmatisation - Discrimination - Prejudice - Stereotype* | - Social stigma - Public opinion* - Public Attitude* - Public Perception* |
