# Supplementary material for: Exploring the stigma experienced by people affected by Parkinson’s disease: a systematic review
Source: BMC Public Health. 2025 Jan 3;25:25. doi: 10.1186/s12889-024-21236-8 (PMC11697948; doi:10.1186/s12889-024-21236-8)
Supplement: Supplementary file 3 — Supplementary Material 3 [file 12889_2024_21236_MOESM3_ESM.docx]

**Identification of new studies via other methods**

**Identification of new studies via databases and registers**

Records identified from*:

Databases (n = 5440)

Registers (n = 0)

Records removed *before screening*:

Duplicate records removed (n = 1190)

Records marked as ineligible by automation tools (n = 0)

Records removed for other reasons (n = 0)

Records identified from:

Websites (n = 0)

Organisations (n = 0)

Citation searching (n = 1)

**Identification**

Reports assessed for eligibility

(n = 46)

Reports sought for retrieval

(n = 46)

Records screened

(n = 4251)

Records excluded**

(n = 4205)

Reports not retrieved

(n = 0)

Reports sought for retrieval

(n = 1)

Reports not retrieved

(n = 0)

**Screening**

Reports excluded: (n = 25)

Wrong outcomes (n = 16)

Wrong population (n = 2)

Wrong intervention (n = 2)

Not empirical research (n = 2)

Systematic/literature review (n = 3)

Reports excluded: (n = 1)

Wrong outcome (n = 1)

Reports assessed for eligibility

(n = 1)

Total studies included in review

(n = 21)

Reports of total included studies

(n = 21)

**Included**

*Consider, if feasible to do so, reporting the number of records identified from each database or register searched (rather than the total number across all databases/registers).

**If automation tools were used, indicate how many records were excluded by a human and how many were excluded by automation tools.

From: Page MJ, McKenzie JE, Bossuyt PM, Boutron I, Hoffmann TC, Mulrow CD, et al. The PRISMA 2020 statement: an updated guideline for reporting systematic reviews. BMJ 2021;372:n71. doi: 10.1136/bmj.n71.
