# Supplementary material for: Exploring the stigma experienced by people affected by Parkinson’s disease: a systematic review
Source: BMC Public Health. 2025 Jan 3;25:25. doi: 10.1186/s12889-024-21236-8 (PMC11697948; doi:10.1186/s12889-024-21236-8)
Supplement: Supplementary file 4 — Supplementary Material 4 [file 12889_2024_21236_MOESM4_ESM.docx]

| **Instrument** | **Measure** | **Validated** | **Studies used** |
| --- | --- | --- | --- |
| Movement Disorder Society – Unified Parkinson’s Disease Rating Scale (MDS-UPDRS) [54] | A 50-question assessment of both motor and non-motor symptoms associated with Parkinson's | Yes | 9  [30, 31, 33-37, 46, 47] |
| Non-motor Symptoms Scale for Parkinson’s Disease (NMSS) [55] | A 30-item rater-based scale to assess a wide range of non-motor symptoms in patients with PD, measuring the severity and frequency of non-motor symptoms across nine dimensions. | Yes | 3  [34-36] |
| Depression, Anxiety and Stress Scale (DASS-21) [56] | A 21-item self-report questionnaire designed to measure the severity of a range of symptoms common to both Depression and Anxiety | Yes | 2  [32, 38] |
| Hamilton Anxiety Rating Scale [57] | A 14-item questionnaire used to measure the severity of anxiety symptoms in adults. | Yes | 2  [34, 36] |
| Hamilton Depression Rating Scale [58] | A 14-item questionnaire used to measure the severity of depression in adults. | Yes | 2  [34, 36] |
| Geriatric Depression Scale (GDS-15) [59] | A 15-item questionnaire used to measure depression in older adults. | Yes | 3  [37, 46, 47] |
