# Supplementary material for: Exploring the stigma experienced by people affected by Parkinson’s disease: a systematic review
Source: BMC Public Health. 2025 Jan 3;25:25. doi: 10.1186/s12889-024-21236-8 (PMC11697948; doi:10.1186/s12889-024-21236-8)
Supplement: Supplementary file 5 — Supplementary Material 5 [file 12889_2024_21236_MOESM5_ESM.docx]

|  | **Aim** | **Country** | **Participants** | **JBI Appraisal Score** | **Data collection method** | **Analysis** | **Key Findings** |
| --- | --- | --- | --- | --- | --- | --- | --- |
| **AboJabel et al (2021)** | To examine the stigmatic experiences of family members of people with PD. | Israel | Spouses/partner of a person with PD (22) | 8 | Focus Groups | Thematic Analysis (Braun & Clarke, 2006) | The study found that spouses of individuals with PD primarily experienced stigma directed at their loved ones rather than themselves as carers. Three major themes emerged: first, public stereotypes of PD, such as associating the disease solely with tremors and old age, leading to frustration and misunderstanding. Second, stigmatising behaviours, including concealing the diagnosis, distancing by family and friends, and disregard by healthcare professionals, were commonly reported. Lastly, structural stigma was evident in social systems, particularly in the lack of support and unequal treatment from institutions like the National Insurance Institute, especially for younger patients. |
|  |  |  | Male – 5 (22%)  Female – 17 (77%)  Other – 0 |  |  |  |  |
|  |  |  | Average Age- 66.76 |  |  |  |  |
| **Caap-Ahlgreen et al (2002)** | To explore women's experiences of living with symptoms related to PD, and to analyse how the symptoms influence their quality of life. | Sweden | People with PD (8) | 7 | Interviews | Phenomenological analysis (Ricoeur, 1997) | The study identified four key themes among women with PD: a wish for a stable body image due to fluctuating physical competence, a desire to maintain traditional female roles despite increasing difficulties with daily tasks, a need for acceptance and reassurance amidst uncertainty, and perceived stigmatisation that leads to social withdrawal. The women expressed feelings of shame, embarrassment, and a preference to be seen as having a handicap rather than PD, which they believe carries a negative reputation. These experiences highlight the significant impact of fluctuating physical and psychosocial competence on their lives. |
|  |  |  | Female – 8 (100%) |  |  |  |  |
|  |  |  | Average Age - 70 |  |  |  |  |
| **Chiong-Rivero et al (2011)** | To assess the perceptions of people living with PD and their carers’ on the impact of PD on HRQOL. | United States of America | People with PD (47) Carers (15) | 7 | Semi-structured interviews  Focus Groups | Thematic Analysis (Braun & Clarke, 2006) | The study identified stigma as a significant concern for people with PD, particularly in social and workplace settings. Patients and caregivers reported feelings of embarrassment and self-stigma due to the physical symptoms of PD, leading to discomfort and unease in their social networks. In the workplace, some patients chose not to disclose their condition to avoid negative reactions. However, not all experiences were negative—some patients found support and kindness from others, challenging their expectations of stigma. These varied experiences highlight the complex emotional impact of living with PD. |
|  |  |  | Male – 24 (38%)  Female – 39 (62%) |  |  |  |  |
|  |  |  | Average Age – 67.1 |  |  |  |  |
| **Chou et al (2024)** | To assess the dyadic dynamics of benefit finding (BF), demoralization, and stigma on the depression severity of PD patients and their caregivers. | Taiwan | People with PD (120)  Carers (120) | 8 | Questionnaires  Interviews | - Descriptive statistics - Chi-square - T-test - Regression model | The stigma and feelings of demoralisation experienced by PD patients and their caregivers may contribute to the severity of depression in these patients. Specifically, the more demoralised and stigmatised both PD patients and their caregivers feel, the more severe the patients' depression tends to be. There are notable interactions between the levels of burden and stigma experienced by both patients and their caregivers. For instance, the degree of burden felt by PD patients and their caregivers significantly influences each other, and similarly, the levels of stigma experienced by both groups also interact in affecting the patients' depression severity. |
|  |  |  | Male – 118 (49.2%) Female – 122 (50.8%) |  |  |  |  |
|  |  |  | Average Age - 63.78 |  |  |  |  |
| **da Silva et al (2020)** | To identify the variables associated with stigma in PD patients who are candidates for deep brain stimulation (DBS). | Brazil | People with PD (54) | 8 | Questionnaires | • Means • Standard deviations (SD) • Median  • Interquartile range • Absolute numbers and frequencies  • The Kolmogorov-Smirnov test  • Simple linear regression  • Multiple linear regression | After conducting a multiple linear regression analysis, it was found that activities of daily living continued to be associated with the stigma experienced by PD patients, with a coefficient of 0.42 (95% CI: 0.003 to 0.83) and a p-value of 0.048. This means that as difficulties with daily activities increased, so did the level of stigma experienced by patients. Overall, the full regression model explained 15% of the variation in the stigma domain, and this relationship was statistically significant (p = 0.03). |
|  |  |  | Male – 34 (63%)  Female – 20 (37%) |  |  |  |  |
|  |  |  | Average Age – 58.2 |  |  |  |  |
| **Eccles et al (2022)** | To investigate the relationships between self-compassion, stigma, and psychological distress among people with PD. | United Kingdom | People with PD (130) | 7 | Questionnaires | • Regressions • Cronbach’s alpha • Spearman’s correlation • Mediation analysis | All variables showed significant correlations. Felt stigma was found to mediate the link between self-compassion and the outcomes of depression, anxiety, and stress. This means that self-compassion affected these outcomes indirectly through its impact on felt stigma. However, self-compassion did not influence the relationship between enacted stigma and distress. This suggests that enacted stigma led to distress independently of how self-compassionate someone was. |
|  |  |  | Male – 43.1 (56%)  Female – 56.9 (74%) |  |  |  |  |
|  |  |  | Average Age - 64 |  |  |  |  |
| **Fothergill-Misbah (2023)** | To explore stigma relating to Parkinson’s in Kenya across the socioecological spectrum, using structural violence as an explanatory concept. | Kenya | People with PD (55)  Carers (23) | 7 | Semi-structured interviews | Thematic Analysis (Braun & Clarke, 2006) | The study highlighted seven key themes related to PD in Kenya: stigma drivers, alternative beliefs about the disease, stigma experiences, and impacts. Stigma stemmed from a lack of awareness and inadequate healthcare resources, compounded by supernatural beliefs and stereotypes that labelled PD as a condition affecting only the "white," "rich," or "old." These misconceptions often led to discrimination, self-stigma, and avoidance of social interactions, exacerbating feelings of isolation and worsening mental and physical health. Some patients faced abandonment due to supernatural beliefs, which led to difficulties accessing care and worsening disease progression. Despite these challenges, some participants found resilience through advocacy and support groups, helping to raise awareness and foster community support. |
|  |  |  | Male – 39 (50%)  Female – 39 (50%) |  |  |  |  |
|  |  |  | Average Age – 66.5 |  |  |  |  |
| **Henry et al (2021)** | To explore cross-cultural differences in the pre- valence of Parkinson’s disease (PD) caregiver affiliate stigma, as well as the relationship between PD symptoms and caregiver affiliate stigma | United States of America and Mexico | Carers (253) | 9 | Questionnaires | Multiple regressions | Multiple regression analyses were conducted to explore how PD symptoms relate to affiliate stigma, and whether this relationship varies by country. The results showed that PD symptoms predict affiliate stigma differently in each country. Specifically, stigma was found to be higher in the US compared to Mexico. Additionally, the link between bowel/bladder symptoms and affiliate stigma was notably stronger in the US. |
|  |  |  | Male 67.9 (26.8%) (total) 32.97 (31.4) (US) 34.9 (23.6) (Mexico)  Female -  185.1 (73.2%) (total) 72.03 (68.6) (US) 113.07 (76.4) (Mexico) |  |  |  |  |
|  |  |  | Average Age – 61.2 |  |  |  |  |
| **Hermanns (2013)** | To explore and describe the perceived stigma shared by 14 participants with PD. | United States of America | Not mentioned | 7 | Semi-structured interviews Observations | Thematic Analysis (Braun & Clarke, 2006) | Participants with PD frequently described feeling isolated, primarily confining themselves to home except for church or medical visits. Stigmatisation stemmed from misperceptions, such as being wrongly labelled as an alcoholic or an "old person's disease," which led to less empathy and support. Visible symptoms like facial masking and communication difficulties contributed to social isolation, while invisible stigma involved psychological impacts and self-concealment. Despite these challenges, participants worked towards self-acceptance and found solace in support groups. One participant's drawing vividly captured their dual experience of feeling 'normal' at home and frail and stigmatised in social settings, highlighting the pervasive impact of stigma on their daily lives. |
|  |  |  | Not mentioned |  |  |  |  |
|  |  |  | Average Age – 68.4 |  |  |  |  |
| **Hou et al (2021)** | To investigate the extent of stigma and its predictive factors in patients with PD from hospital | China | People with PD (276) | 8 | Questionnaires | • Multivariate linear regression  • Kolmogorov-Smirnov test • Absolute numbers • Median • Frequencies • Pearson’s correlation • Spearman’s correlation | The average score for social stigma (SSCI) among participants was 49.9, with felt stigma averaging 29.4 and enacted stigma at 20.6. About 48.5% of patients reported experiencing stigma "rarely" to "sometimes." Analysis revealed that factors such as age, disease duration, cognitive function, non-motor symptoms, depression, anxiety, motor symptoms, and specific subtypes of motor symptoms (tremor-dominant and postural instability/gait difficulty) were significantly associated with social stigma. Multivariate linear regression indicated that motor symptoms (UPDRS III), the tremor-dominant subtype, depression, disease duration, and age independently predicted social stigma, accounting for 47.8% of its variance. Gender differences showed that in females, felt stigma was linked to higher motor symptoms and depression, while in males, it related to higher motor symptoms, longer disease duration, and depression. Enacted stigma in females was associated with higher motor symptoms and younger age, whereas in males, it was associated with the tremor-dominant subtype and motor symptoms. |
|  |  |  | Male – 135 (48.7%)  Female – 141 (50.9%) |  |  |  |  |
|  |  |  | Average Age – 62.5 |  |  |  |  |
| **Islam et al (2022)** | To assess the impact of health conditions on perceived stigma and Quality of Life (QoL) in persons with PD. | United States of America | People with PD (196) | 8 | Questionnaires | • T-test • Point-biseral correlation • Analysis of covariance (ANCOVA) | Among the participants, 79% reported having at least one additional health condition alongside PD, such as thyroid disease, headache, and heart disease. The presence of multiple health conditions was linked to an increased perception of stigma and a lower QoL, with particular associations observed for thyroid disease, depression, and anxiety. Additionally, younger individuals, those with less education, and those diagnosed with PD at a younger age experienced higher levels of stigma. This suggests that both the complexity of managing multiple health issues and individual factors such as age and educational background play significant roles in how stigma is perceived and how it impacts quality of life in those with PD. |
|  |  |  | Male – 81 (41.3%)  Female – 113 (57.6%) |  |  |  |  |
|  |  |  | Average Age – 64.8 |  |  |  |  |
| **Lin et al (2022)** | To investigate the development and evolution of self-stigma in patients with early stage PD and to explore the associated and predictive factors of self-stigma in PD. | China | People with PD (224) | 9 | Questionnaires | • Mean • Standard deviation • T-test • Benjamini-Hochberg procedure • Multiple linear regression • Generalised estimating equation • Binary logistic model • Multivariate logistic model | The study found that self-stigma was commonly experienced by patients with PD, particularly during the early stages of the disease. Interestingly, there was no significant relationship between motor symptoms and levels of self-stigma. Over time, self-stigma was observed to decrease as the disease progressed, marking it as a nonpersistent and diminishing issue. This is the first study to reveal that self-stigma in PD is reversible, suggesting that it can diminish as patients adapt to their condition. Notably, depression was identified as the sole associated and predictive factor for self-stigma, indicating that addressing depressive symptoms could be an effective strategy for reducing self-stigma in individuals with PD. |
|  |  |  | Male – 121 (54%)  Female – 103 (45.9%) |  |  |  |  |
|  |  |  | Average Age – 58.45 |  |  |  |  |
| **Ma et al (2019)** | To examine the relationship between self-reported facial masking and quality of life in people with PD To test experienced stigma as a mediator and gender as a moderator of this relationship | United States of America | People with PD (90) | 5 | Questionnaires | • Descriptive statistics • Key variable correlation coefficient • Chi-square • T-test • Ordinary least squares regression • Simple mediation analyses • Parallel multiple mediation analysis  • Conditional process analysis • Pearson’s correlation | Individuals with PD reported greater difficulty with facial expressions tended to experience higher levels of stigma, which significantly impacted their QoL. This relationship was meaningful, underscoring the substantial effect that challenges with facial masking can have on personal well-being. Notably, for women, each additional point of facial masking was associated with a more pronounced increase in perceived stigma compared to men. Consequently, this stronger link between facial masking and experienced stigma contributed to a slightly greater reduction in QoL for women with PD. |
|  |  |  | Male – 56 (62%)  Female – 34 (38%) |  |  |  |  |
|  |  |  | Average Age – 65.49 |  |  |  |  |
| **Ma et al (2016)** | To examine the role of experienced stigma in health-related quality of life (QOL), after controlling for personal and clinical characteristic. | United States of America | People with PD (73) | 7 | Questionnaires | • Descriptive statistics • Pearson’s correlation • Multiple regression | Stigma emerges as a crucial factor influencing the QoL for individuals with PD. Those who reported experiencing stigma were often found to have more severe symptoms of PD, including greater motor difficulties and more pronounced depressive symptoms, all of which contributed to a lower QoL. People living with PD expressed significant concern about the stigma they face, with felt stigma—where individuals perceive or anticipate being judged—being more commonly experienced than enacted stigma, which involves actual negative behaviour from others. Felt stigma has a particularly strong impact on QoL, affecting individuals' mental health and overall life satisfaction. Consequently, participants who encountered stigma tended to exhibit higher levels of depression and report poorer QoL, highlighting the profound effect stigma can have on their well-being. |
|  |  |  | Male – 44 (60.3%)  Female – 29 (39.7%) |  |  |  |  |
|  |  |  | Average Age – 65.72 |  |  |  |  |
| **Mshana et al (2011)** | The aims of this study were to investigate the experience and treatment seeking behaviours of PD sufferers and their carers together with community understandings of PD in a rural part of Tanzania. | Tanzania | People with PD (n=28) Carers (n=28) Health Workers (n=4) Traditional Healers (n=2) | 8 | Semi-structured interviews  Focus Groups | Thematic Analysis (Braun & Clarke, 2006) | Some participants reported experiencing stigma related to PD from family and community members, including beliefs that PD was caused by curses or witchcraft, leading some to seek ineffective traditional treatments. Despite some understanding that PD primarily affects the elderly, misconceptions persisted, such as associating PD with old age, cold weather, or supernatural causes. This lack of awareness and incorrect beliefs contributed to social isolation and hindered timely and appropriate care for those affected by PD. |
|  |  |  | Male – 32 (52%)  Female – 30 (48%) |  |  |  |  |
|  |  |  | Average Age – 69.5 |  |  |  |  |
| **Nazzal & Khalil (2016)** | To explore the lived experiences of Jordanian individuals with PD and how does the disease affect their daily life. | Jordan | People with PD (8) | 8 | Semi-structured interviews | Thematic Analysis (Braun & Clarke, 2006) | All participants reported experiencing stigma related to PD including being stared at and laughed at for their involuntary movements. This stigma equally affected both men and women, leading to depression and anxiety. Younger individuals and those newly diagnosed with PD faced more severe stigmatising attitudes compared to older individuals with long-term diagnoses. Stigma led many to socially isolate themselves, hide their disability, and avoid sharing their diagnosis, often leading to significant psychological distress and reduced social participation. Consequently, participants, especially women, reported limiting their social activities and experiencing negative impacts on their daily lives due to fear of stigma and mobility issues. |
|  |  |  | Male – 4 (50%)  Female – 4 (50%) |  |  |  |  |
|  |  |  | Average Age - 57 |  |  |  |  |
| **Parry et al (2022)** | To examine the experience of people with Parkinson’s disease when walking in different social situations, and improve understanding of how this affects participation in meaningful activity. | France | People with PD (14) | 7 | Semi-structured interviews  Observations | Thematic Analysis (Braun & Clarke, 2006) | Participants with PD frequently reported distress over being stared at due to their distinctive gait and involuntary movements, describing it as one of the most challenging aspects of their condition. Younger individuals, in particular, felt the sting of stigma more acutely as movement disorders are often associated with old age. This scrutiny led many to avoid public spaces, conceal their symptoms, or use mobility aids to manage perceptions. Social settings amplified the impact of being looked at, with participants feeling more self-conscious and distressed in public. Despite some efforts to improve self-image and manage public perceptions, stigma and self-consciousness about their appearance often led to social isolation and avoidance of social interactions. |
|  |  |  | Male – 11 (79%)  Female – 3 (21%) |  |  |  |  |
|  |  |  | Average Age - 61 |  |  |  |  |
| **Salazar et al (2019)** | To assess the contributions of clinical and demographic characteristics to self-perceived stigma in a PD sample that was in the early to middle stages of the disease, when potential self-perceived stigma may first be appearing. | United States of America | People with PD (362) | 5 | Questionnaires | • Mean • Standard deviation • Pearson’s correlation • T-test • Hierarchical regression analysis • Regression models • Linear regression • Mediation analysis | Depression and younger age emerged as the primary predictors of self-perceived stigma in individuals with PD. For both men and women, higher levels of depression were strongly associated with increased self-perceived stigma, while younger age was a significant predictor only for men. Disease characteristics, such as the severity or duration of PD, did not significantly influence stigma perception. The full model explained 14% of the variance in stigma perception, with both younger age and higher depression scores being significant predictors. Specifically, in men, both factors were important, whereas in women, depression alone was the key predictor. Additionally, depression was found to mediate the relationship between stigma and difficulties in activities of daily living, underscoring its critical role in the experience of stigma among PD patients. |
|  |  |  | Male – 205 (56.6%)  Female – 157 (43.3%) |  |  |  |  |
|  |  |  | Average Age - 67 |  |  |  |  |
| **Shah et al (2022)** | To explore the views and experiences of how people living with Parkinson’s self-manage their condition and identify areas needed to be incorporated into self-management resources or interventions. | United Kingdom | People with PD (20) | 7 | Semi-structured interviews | Thematic Analysis (Braun & Clarke, 2006) | Under the theme of "barriers to self-management," societal attitudes and stigma around PD significantly impact participants' confidence and self-care. Many reported that widespread misunderstanding and negative perceptions of PD, especially among younger individuals, led to embarrassment and avoidance of public spaces. Feelings of low self-esteem and isolation were common, with some participants even concealing their condition from others, including at work. Cultural views also played a role, with one participant noting that cultural stigma in their home country led to preferring to be mistaken for a drunk rather than openly acknowledging their PD. |
|  |  |  | Male – 12 (60%)  Female – 8 (40%) |  |  |  |  |
|  |  |  | Average Age - 72 |  |  |  |  |
| **Valcarenghi et al (2017)** | To understand the daily lives of people with Parkinson’s disease. | Brazil | People with PD (30) | 7 | Semi-structured interviews | Thematic Analysis (Braun & Clarke, 2006) | The theme "living with the stigma" reveals how prejudice against PD arises from its physical symptoms, such as tremors and altered gait, leading to embarrassment and shame. Participants described feeling pitied or stared at in public, which often results in avoiding social interactions and hiding their symptoms. This stigma can diminish self-esteem and confidence, as individuals with PD experience discomfort from others' reactions and misconceptions about their condition. Additionally, there is a perceived lack of appropriate healthcare support for younger patients, who feel neglected by programmes typically focused on older adults, reinforcing the misconception that PD primarily affects the elderly. |
|  |  |  | Male – 11 (37%)  Female – 19 (63%) |  |  |  |  |
|  |  |  | Average Age – 63.5 |  |  |  |  |
| **Verity et al (2020)** | To investigate whether the perception of control mediates the relationship between stigma and well-being in people with Parkinson’s disease. | United Kingdom | People with PD (229) | 8 | Questionnaires | • Inferential Analysis • Spearman’s correlation • Mediation regression • Correlation analysis • Mediation analysis | Perceived control significantly mediates the impact of stigma on Health-Related QoL, depression, and positive affect. This means that how much control individuals feel they have can influence how stigma affects their overall well-being, mood, and outlook. However, perceived control does not mediate the relationship between stigma and anxiety or stress, indicating that other factors might be more influential in these areas. Even with perceived control accounted for, the direct effects of stigma on HRQoL, depression, and positive affect remain significant. In practical terms, while stigma negatively impacts these aspects of well-being, perceived control adds a layer of influence but does not fully explain the relationship between stigma and anxiety or stress. |
|  |  |  | Male – 113 (49%)  Female – 116 (51%) |  |  |  |  |
|  |  |  | Average Age - 65 |  |  |  |  |
| **Warren et al (2016)** | To explore the experience of diagnosis in people with PD with a relatively recent diagnosis (in the last 1–18 months). | United Kingdom | People with PD (6) | 7 | Semi-structured interviews | Thematic Analysis (Braun & Clarke, 2006) | The themes highlight two key aspects of living with PD. First, a lack of understanding and skewed public perceptions, often attributing PD symptoms to old age or clumsiness, contribute to misinterpretations and diminished recognition of early-stage symptoms. Second, the social implications of a PD diagnosis involve significant challenges with accepting help and dealing with the stigma of losing independence and employment. Participants reported struggling with the loss of their job and the negative societal views of their condition, which further complicate their experience of the disease. |
|  |  |  | Male – 5 (83%)  Female – 1 (17%) |  |  |  |  |
|  |  |  | Average Age – 74.6 |  |  |  |  |
